# Risk factors associated with poorer experiences of end-of-life care and challenges in early bereavement: Results of a national online survey of people bereaved during the COVID-19 pandemic

**DOI:** 10.1177/02692163221074876

**Published:** 2022-02-17

**Authors:** Lucy Ellen Selman, DJJ Farnell, M Longo, S Goss, K Seddon, A Torrens-Burton, CR Mayland, D Wakefield, B Johnston, A Byrne, E Harrop

**Affiliations:** 1 Palliative and End of Life Care Research Group, Population Health Sciences, Bristol Medical School, University of Bristol, Bristol, UK; 2 School of Dentistry, Cardiff University, Cardiff, UK; 3 Marie Curie Research Centre, Cardiff University, Cardiff, UK; 4 Wales Cancer Research Centre, Cardiff, UK; 5 Department of Oncology and Metabolism, University of Sheffield, Sheffield, UK; 6 North Tees and Hartlepool NHS Foundation Trust, Stockton-on-Tees, UK; 7 School of Medicine, Dentistry and Nursing, University of Glasgow, Glasgow, UK

**Keywords:** Grief, pandemics, bereavement, coronavirus infections, bereavement services, terminal care, palliative care

## Abstract

**Background:**

Experiences of end-of-life care and early bereavement during the COVID-19 pandemic are poorly understood.

**Aim:**

To identify clinical and demographic risk factors for sub-optimal end-of-life care and pandemic-related challenges prior to death and in early bereavement, to inform clinical practice, policy and bereavement support.

**Design:**

Online national survey of adults bereaved in the UK (deaths between 16 March 2020 and 2 January 2021), recruited via media, social media, national associations and organisations.

**Setting/participants:**

711 participants, mean age 49.5 (SD 12.9, range 18–90). 628 (88.6%) were female. Mean age of the deceased was 72.2 (SD 16.1, range miscarriage to 102 years). 311 (43.8%) deaths were from confirmed/suspected COVID-19.

**Results:**

Deaths in hospital/care home increased the likelihood of poorer experiences at the end of life; for example, being unable to visit or say goodbye as wanted (*p* < 0.001). COVID-19 was also associated with worse experiences before and after death; for example, feeling unsupported by healthcare professionals (*p* < 0.001), social isolation/loneliness (OR = 0.439; 95% CI: 0.261–0.739), and limited contact with relatives/friends (OR = 0.465; 95% CI: 0.254–0.852). Expected deaths were associated with a higher likelihood of positive end-of-life care experiences. The deceased being a partner or child also increased the likelihood of positive experiences, however being a bereaved partner strongly increased odds of social isolation/loneliness, for example, OR = 0.092 (95% CI: 0.028–0.297) partner versus distant family member.

**Conclusions:**

Four clear risk factors were found for poorer end-of-life care and pandemic-related challenges in bereavement: place, cause and expectedness of death, and relationship to the deceased.

**What is already known about the topic?**

- Since the start of the pandemic, it is estimated that over 50 million family members and friends have been bereaved due to COVID-19, with millions more bereaved due to other causes.
- Bereavement of any cause during the COVID-19 pandemic is associated with specific challenges, including limited access to people before their death, pressure on health and social care providers, quarantining due to infection or exposure, lockdowns and social distancing.
- No previous studies have identified factors associated with sub-optimal end-of-life care or challenging experiences in bereavement, and this evidence is needed to inform clinical practice, bereavement support and policy.

**What this paper adds?**

- Our study highlights four risk factors for poorer end-of-life care and increased risk of pandemic-related challenges in early bereavement: place, cause and expectedness of death and relationship to the deceased.
- COVID-19 deaths, hospital and care home deaths and unexpected deaths were generally associated with poorer outcomes, while being a partner of the person who died (regardless of cause) and bereavement due to COVID-19 increased the odds of experiencing social isolation and loneliness in bereavement.

**Implications for practice, theory or policy**

- Partners bereaved due to an unexpected COVID-19 death in hospital may be at particular risk of poor outcomes and need additional follow-up and support.
- Deaths in hospitals and care homes can be improved by prioritising communication with and involvement of relatives, facilitating contact between loved ones prior to death as much as possible and improving sign-posting to bereavement support.
- Clear and consistent national guidance on hospital, hospice and care home visiting and prioritising personal protective equipment for end-of-life care providers is essential in a pandemic to ensure equity, support staff and improve experiences among bereaved people.

## Background

The COVID-19 pandemic has resulted in widespread, mass bereavement on an unprecedented global scale, with 5.6 million deaths from COVID-19 recorded so far.^1^ With each death associated with approximately nine close bereavements,^2^ an estimated 50.4 million family members and friends have been bereaved due to COVID-19 since the start of the pandemic. Deaths during the pandemic are associated with risk factors for poor bereavement outcomes identified pre-pandemic, including traumatic end-of-life and death experiences, being unable to say goodbye, loss of community networks and social support, and social and economic disruption.^3–9^ Many of these challenges are relevant to non-COVID-19 deaths as well as bereavements due to COVID-19.

In the UK, infection control advice was first issued by the government on 16 March 2020. End-of-life care and infection control restrictions such as social distancing policies have varied since the start of the pandemic, with inconsistencies across geographical areas and care settings. Recent research has described the challenges of providing end-of-life-care during the pandemic^10^ and qualitatively investigated bereavement experiences^11^ and support needs.^12^ However, there remains little evidence to inform optimal clinical practice, bereavement support and the policy response to COVID-19 as a mass bereavement event. This study aimed to identify clinical and demographic risk factors associated with experiencing sub-optimal end-of-life care or pandemic-related challenges prior to death and in early bereavement, using baseline data from a mixed-methods longitudinal study of bereavement during the pandemic in the UK.^12,13^

## Methods

### Design

An open web survey (Supplemental material 1), disseminated to a convenience sample of people bereaved during the pandemic in the UK.

### Survey development

Survey items and structure were informed by study aims and previous research.^14–17^ The survey was designed with input from the advisory group and piloted, refined and tested with members of the public. Non-randomised open and closed questions covered end-of-life and grief experiences, and perceived needs for, access to and experiences of bereavement support.

### Primary outcomes

#### Experiences of end-of-life care

Six items, adapted from the Consumer Quality Index for Palliative Care,^14^ assessed end-of-life care experiences: involvement in care decisions, knowing the contact details for the professional responsible for care, receiving information about the approaching death, support by healthcare professionals immediately after the death, contact by the hospital/care provider after the death, and provision of information about bereavement support services. These items were developed through research with relatives to assess the quality of palliative care, and have been psychometrically tested and found to be reliable and/or of high importance to relatives.

#### Pandemic-related challenges

Six items assessed pandemic-related challenges prior to and after the death. Exploratory factor analysis found two subscales, related to problems due to ‘contact with loved one prior to the death’ (unable to visit prior to death, limited contact in last days of life, unable to say goodbye) and ‘social isolation’ (restricted funeral arrangements, social isolation and loneliness, limited contact with others). All items were answered yes/no. For each participant, the number of problems experienced in each subscale and the total number of problems overall were calculated by summing item scores (0 = no and 1 = yes; possible range: 0–3 for each subscale, 0–6 for total). Cronbach’s α was 0.57, 0.57 and 0.64 for the ‘contact prior to death’ subscale, ‘social isolation’ subscale and total respectively. These values for Cronbach’s α indicate adequate levels of reliability/internal consistency.

### Associated factors

We assessed whether demographic and clinical factors independently predicted end-of-life care experiences and pandemic-related challenges. Factors included in the analysis are recognised risk factors for poor bereavement outcomes (age, gender, relationship to deceased, expectedness of the death)^7,18^ or have been identified as indirectly associated with experiences of end-of-life care (qualifications, deprivation level and region; place of death; cause of death).^19,20^ We used postcode data to identify geographical region of residence and (for England) socio-economic deprivation.

### Study procedure

The voluntary survey was administered via Jisc software^21^ and was open from 28th August 2020 to 5th January 2021. It was disseminated to a convenience sample on social and mainstream media and via voluntary sector associations and bereavement support organisations, including those serving ethnic minority communities. These organisations helped disseminate the survey by sharing on social media and other forums and via direct invitations to potential participants (see Supplemental material 2 for example advertisement). For ease of access, the survey was posted onto a bespoke study-specific website with a memorable URL.^22^ Two participants chose to complete the survey in paper format. Summaries of survey results (including interim results released November 2020) were posted on the study website and provided to interested participants.

Inclusion criteria: aged 18+; family or close friend bereaved since social-distancing requirements were introduced in the UK (16/03/2020); death occurred in the UK; ability to consent. Anyone not meeting these criteria was excluded from participating. The initial section of the survey requested informed consent and details of data protection (Supplemental material 1).

Reporting follows the Checklist for Reporting Results of Internet E-Surveys.^23^

### Data analysis

A statistical analysis plan was drafted by the study statistician (DJJF) and refined iteratively by all members of the research team. The main research questions explored in this paper were: how common were pandemic-related end of life and social issues such as lack of contact, disrupted mourning rituals and isolation, and what were the most important factors that affected them?

Frequency tables were used to explore the data. A Directed Acyclic Graph was also used to visualise the relationships between variables and generate hypotheses for statistical testing in both univariate calculations and mixed-effects logistic regression via a mixed-effects generalised linear model (GLM). Cohen’s *h* effect size effect was used to measure the differences between two proportions. For any comparisons, the maximum value of *h* is presented to provide an estimate of (maximum) effect size (*h* = 0.2: small effect, *h* = 0.5: medium effect, *h* ⩾ 0.8: large effect). Chi-squared tests and Fisher’s exact test were used for categorical outcome data. GLM was used to examine pandemic-specific challenges as a function of factors identified as having a strong and/or significant effect on outcomes, as derived from Cohen’s *h* effect sizes and univariate calculations. Region of the UK was included as a random effect, whereas all other variables were coded as fixed effects. Results of the mixed-effects GLM analysis are presented as odds ratios in supplemental tables. To provide a comparison to the results from the mixed-effects GLM, odds ratios were also found via simple logistic regression. All calculations were carried out using SPSS V26.

### Ethical approvals

The study protocol and supporting documentation was approved by Cardiff University School of Medicine Research Ethics Committee (SMREC 20/59). The study was conducted in accordance with the Declaration of Helsinki and all respondents provided informed consent.

## Results

### Sample characteristics

711 bereaved people participated (Table 1). 12 surveys were completed in duplicate; the first survey was retained for these participants. Two surveys were excluded as only the consent question had been answered. Missing data was minimal (mean per item 0.7%, range 0%–3.7%). Imputation of missing data was not necessary.

**Table 1.** Characteristics of the bereaved person.

Age			
Age (years)	Mean [Median]	SD	Min–Max
	49.5 [50.0]	12.9	18–90
	*n*	Percentage	
Gender identity			
Male	74	10.4%	
Female	628	88.6%	
Other	7	1.0%	
Ethnicity			
Non-BAME (total)	676	95.3%	
White British	438	64.8%	
White English	111	16.4%	
White Welsh	41	6.1%	
Northern Irish	22	3.3%	
White Scottish	40	5.9%	
Any other white	17	2.5%	
White Irish	7	1.0%	
BAME (total)	33	4.7%	
White and Black Caribbean	12	36.4%	
White and Asian	5	15.2%	
Indian	4	12.1%	
Black Caribbean	4	12.1%	
Any other mixed background	3	9.1%	
Pakistani	1	3.0%	
Bangladeshi	1	3.0%	
Arab	1	3.0%	
White and Black African	1	3.0%	
Any other Asian	1	3.0%	
Religious beliefs			
Buddhism	8	1.2%	
Christian	251	36.7%	
Hinduism	3	0.4%	
Islam	5	0.7%	
Judaism	6	0.9%	
Sikhism	2	0.3%	
Other or agnostic	107	15.7%	
No	301	44.1%	
Highest qualification			
None or GCSEs	108	15.3%	
A-level or Apprenticeship or ONC	132	18.6%	
HND or University Degree	468	66.1%	
Region			
England	517	78.5%	
Wales	63	9.6%	
Scotland	53	8.0%	
Northern Ireland	26	3.9%	
Unemployed during the pandemic?			
Yes	55	7.9%	
No	645	92.1%	
Bereavements in previous year?			
Yes	158	22.5%	
No	543	77.5%	
IMD Decile (England only) (*n* = 517)			
1	26	5.0%	
2	45	8.7%	
3	49	9.5%	
4	52	10.1%	
5	64	12.6%	
6	52	10.1%	
7	58	11.2%	
8	57	11.0%	
9	46	8.9%	
10	50	9.7%	

BAME: Black, Asian or minority ethnic background; GCSE: General Certificate of Secondary Education for 15 and 16 year olds in the UK; A Levels: Advanced Level subject-based qualification for students in the UK aged 16 and above; ONC: Ordinary National Certificate (equivalent to A Levels); HND: Higher National Diploma (vocational qualification provided by higher or further education colleges in the UK); IMD: indices of multiple deprivation.

Participants represented diverse geographical areas, deprivation indexes and levels of education. 628 (88.6%) participants were female; the mean age was 49.5 years (SD = 12.9; range 18–90). 395 (55.6%) participants had experienced the death of a parent, followed by partner/spouse (*n =* 152, 21.4%). 72 people (10.1%) had experienced more than one bereavement since 16th March 2020. 33 people (4.7%) self-identified as from a minority ethnic background.

The mean age of the deceased person was 72.2 years (SD = 16.1; range: miscarriage at 4 months to 102 years’ old) (Table 2). 311 (43.8%) died of confirmed/suspected COVID-19, 156 (21.9%) from cancer, and 119 (16.7%) from another life-limiting condition. Most died in hospital (*n =* 410; 57.8%). Questionnaires were completed a median of 152 days (5 months) after the death (range 1–279 days).

**Table 2.** Characteristics of the deceased.

Age (years)	Mean [Median]	SD	Min–Max
	72.2 [74.0]	16.1	0–102
	*n*	Percentage	
Relationship of the deceased person to the bereaved*			
Partner (Male/Female)	152 (129/23)	21.4% (18.1%/3.2%)	
Parent (Father/Mother)	395 (218/197)	55.6% (30.7%/27.7%)	
Grandparent	54	7.6%	
Sibling (Brother/Sister)	23 (15/10)	3.2% (2.1%/1.4%)	
Child (Son/Daughter)	15 (12/4)	2.1% (1.7%/0.6%)	
Other family member	46	6.5%	
Colleague or friend	26	3.7%	
Cause of death			
COVID	273	38.5%	
Suspected COVID	38	5.4%	
Non-COVID (total)	399	56.2%	
Cancer	156	21.9%	
Other PLLC**	118	16.7%	
Non-PLLC/SD***	112	15.8%	
Don’t know	12	3.0%	
Not specified	1	0.2%	
Was the death expected?			
Yes	113	16.0%	
No	552	78.0%	
Don’t know	43	6.1%	
Place of death			
In hospital	410	57.7%	
In their home	158	22.2%	
In a hospice	37	5.2%	
In a care home	91	12.8%	
Other/Don’t know	13	1.8%	

* Multiple bereavements recorded by participants explain discrepancies between overall totals in sibling, child and parent groups and their sub-categories.

** PLLC: progressive life-limiting condition; for example, heart disease, COPD, dementia.

*** Non-PLLC/SD: non-progressive life-limiting condition or sudden death; for example, stroke, heart attack, accident, suicide.

### Main outcomes

#### End-of-life care experiences

There was wide variation in overall experiences of end-of-life care (Table 3); for example, while 21.8% reported they were always involved in decision about their friend or relative’s care, 21.8% reported that they were never involved; 32.3% reported that they were fully informed about the approaching death while 17.7% said they were not at all informed. Half the participants (49.8%) knew the contact details for the professional responsible for their friend or relative’s care. 28.2% reported that they were very or fairly well supported by professionals immediately after the death, while 35.4% felt not at all supported. Overall, a third (34%) reported that a professional had provided information about bereavement support services. Between 11.7% and 19.7% of respondents answered ‘not relevant’ to these questions, for example, because they were not next of kin, or no healthcare providers were involved in the death.

**Table 3.** Frequency of end-of-life care experiences.

		*N*	%
Did the care professionals involve you in decisions about the care for your sick loved one?	Never	155	21.8
	Sometimes	162	22.8
	Usually	98	13.8
	Always	155	21.8
	Not relevant to my situation (e.g. not next of kin, because none were involved)	140	19.7
	Missing	1	0.1
Did you know the contact details for the professional responsible for their care?	Yes	354	49.8
	No	193	27.1
	Not sure	52	7.3
	Not relevant to my situation	109	15.3
	Missing	3	0.4
Did you receive information about the approaching death?	No, not at all	126	17.7
	A bit of information	270	38.0
	Yes, I was fully informed	230	32.3
	Not relevant to my situation	83	11.7
	Missing	2	0.3
Did you feel well supported by the healthcare professionals immediately after the death of your loved one?	Very well supported	95	13.4
	Fairly well supported	105	14.8
	A little bit supported	139	19.5
	Not at all supported	252	35.4
	Not relevant to my situation (e.g. because none were involved or not next of kin)	120	16.9
Were you contacted again by the hospital or care provider following their death?	Yes	251	35.3
	No	322	45.3
	Not relevant to my situation	138	19.4
Did they provide information about bereavement support services?	Yes (at the time of death)	131	18.4
	Yes (during follow up call)	89	12.5
	Yes (at the time of death and during follow up call)	22	3.1
	No	342	48.1
	Not relevant to my situation	119	16.7
	Missing	8	1.1

#### Pandemic-related challenges

Participants reported a mean of 4.17 (median = 4) pandemic-related problems (out of a maximum of 6) (Table 4). People reported significantly higher levels of problems due to social isolation (mean = 2.41, median = 3) than problems related to contact before death (mean = 1.76, median = 2; Wilcoxon signed-rank test: *z* = 12.344, *p* < 0.001). The three most prevalent items were restricted funeral arrangements (93.4%), limited contact with other close relatives/friends (80.7%) and social isolation and loneliness (66.7%).

**Table 4.** Frequency of pandemic-related challenges before or after the death.

Subscale	Item	Percentage (95% CI)*
Contact prior to death	Unable to visit them prior to their death	54.3% (50.5%–58.0%)
	Limited contact with them in last days of their life	57.8% (54.1%–61.5%)
	Unable to say goodbye as I would have liked	63.9% (60.2%–67.4%)
Social isolation	Restricted funeral arrangements	93.4% (91.3%–95.1%)
	Social isolation and loneliness	66.7% (63.1%–70.1%)
	Limited contact with other close relatives or friends	80.7% (77.6%–83.6%)

* Note that percentages are with respect to those participants who responded ‘yes’ to these items.

### Overview of the influence of important factors on the outcomes

**Figure 1.**
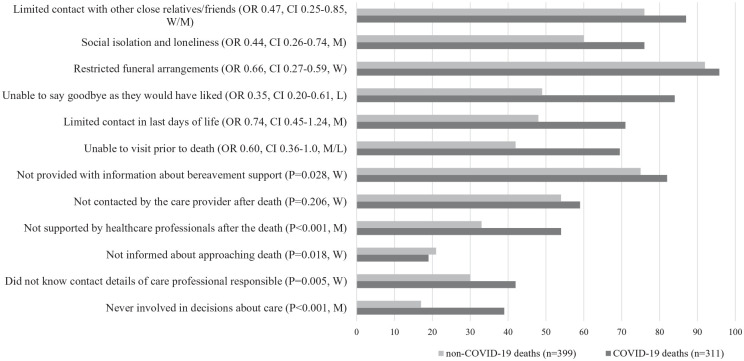
Proportion of poor end-of-life and bereavement experiences for COVID-19 and non-COVID deaths (%). OR is for mixed-effects generalised linear model; 95% CIs; W: weak effect (Cohen’s *h* = 0.2); M: medium effect (Cohen’s *h* = 0.5); L: large effect (Cohen’s *h* = 0.8).

Effect sizes measured via Cohen’s *h* were used to determine those factors and covariates that had the strongest influence on the outcome measures and we describe the effects of these factors on the outcomes in detail below. Table 5 and Figure 1 provide an overview of these results. Poorer end-of-life care experiences were associated mostly strongly with: deaths in hospital/care home compared with other places of death, deaths due to COVID-19 compared with non-COVID deaths and unexpected deaths compared with those that were expected. Closer relationships (especially the deceased being a partner or child) compared with more distant relationships (distant family/colleague or friend) were associated with better end-of-life care experiences, however social isolation and loneliness was highest for bereaved partners. For completeness, the effects of other demographic factors on the outcomes are also described below, although they were found to have a smaller effect on the outcomes.

**Table 5.** Pandemic-related challenges compared by: relationship to deceased, place of death, cause of death and unexpectedness of death. Note that percentages are with respect to those participants who responded ‘yes’ to the pandemic-related challenge items. Effect sizes are estimated from the maximum Cohen’s *h* between any two groups for a given factor, where: *h* = 0.2: small effect, h = 0.5: medium effect, *h* ⩾ 0.8: large effect.

	*n*	Unable to visit them prior to their death	Limited contact with them in last days of their life	Unable to say goodbye as I would have liked	Restricted funeral arrangements	Social isolation and loneliness	Limited contact with other close relatives or friends
Partners	152	41.4%	46.1%	49.3%	94.7%	81.6%	80.9%
Parents	395	52.7%	63.3%	65.3%	94.4%	65.1%	82.8%
Grandparents	54	77.8%	63.0%	77.8%	90.7%	57.4%	75.9%
Sibling	23	65.2%	56.5%	73.9%	87.0%	56.5%	73.9%
Child	15	33.3%	26.7%	53.3%	93.3%	66.7%	73.3%
Other family member	46	80.4%	63.0%	80.4%	93.5%	52.2%	82.6%
Colleague or friend	26	61.5%	42.3%	65.4%	80.8%	57.7%	65.4%
(Maximum) Cohen’s *h*		1.19 (Large)	0.83 (Large)	0.84 (Large)	0.61 (Medium)	0.81 (Large)	0.53 (Medium)
Chi-squared test: *p*		<0.001	0.001	<0.001	0.12	<0.001	0.307
Died in hospital	410	63.7%	66.1%	73.7%	93.9%	68.8%	82.4%
Died in their home	158	29.7%	32.3%	39.2%	92.4%	63.9%	77.8%
Died in a hospice	37	43.2%	43.2%	32.4%	100.0%	75.7%	89.2%
Died in a care home	91	64.8%	76.9%	80.2%	90.1%	63.7%	78.0%
Other/Don’t Know	13	15.4%	15.4%	30.8%	92.3%	38.5%	61.5%
(Maximum) Cohen’s *h*		1.10 (Large)	1.45 (Large)	1.24 (Large)	0.90 (Large)	0.93 (Large)	0.88 (Large)
Chi-squared test: *p*		<0.001	<0.001	<0.001	0.311	0.102	0.143
COVID	311	69.8%	70.7%	83.6%	95.5%	75.6%	86.8%
Non-Covid	399	42.4%	47.9%	48.6%	91.7%	59.9%	75.9%
(Maximum) Cohen’s *h*		0.67 (Medium/Large)	0.57 (Medium)	0.96 (Large)	0.22 (Small)	0.43 (Medium)	0.38 (Small/Medium)
Fisher’s exact text: *p*		<0.001	<0.001	<0.001	0.049	<0.001	<0.001
Expected loved one to die	113	31.0%	35.4%	38.1%	88.5%	59.3%	77.0%
Did not expect loved one to die	552	59.6%	62.1%	70.1%	94.2%	69.2%	81.7%
Don’t know	43	48.8%	60.5%	51.2%	95.3%	55.8%	79.1%
(Maximum) Cohen’s *h*		0.65 (Medium/Large)	0.62 (Medium)	0.77 (Large)	0.36 (Small/Medium)	0.34 (Small/Medium)	0.16 (Small)
Chi-squared test: *p*		<0.001	<0.001	<0.001	0.073	0.036	0.484

### Place of death

Place of death had a moderate to strong influence on end-of-life care outcomes (Tables S1–S7). If the death occurred in a hospice or at home the bereaved were more likely to be involved in decisions about the care for their friend or relative (*p* < 0.001) and feel well supported by healthcare professionals immediately after the death (*p* = 0.003) than if the death occurred in a hospital or care home (Tables S1 and S4). If the person had died in hospital the bereaved was less likely to know the contact details for the professional responsible for their friend or relative’s care (*p* = 0.001) compared with other settings. Where the death had occurred in a hospice the bereaved person was most likely to have been provided with information about bereavement support services both at the time of death and during a follow-up call, while this was least likely for care home deaths (*p* < 0.001).

The effect size for place of death was high across all pandemic-related challenges. When a person had died in hospital or in a care home participants were most likely to report problems for all items in the ‘contact with loved one prior to death’ subscale (*p* < 0.001) compared with other places of death (Table 5). Mixed-effects GLM analyses showed clearly that when death occurred at home, in a hospice or ‘other/don’t know’ the participant had strongly decreased odds of being unable to visit their friend or relative before death, limited contact with them in last days of their life and being unable to say goodbye as they would have liked compared with death in hospital (Tables S8–S10). Differences did not reach statistical significance for restricted funeral arrangements, social isolation and loneliness, and limited contact with other close relatives/friends (Table 5; Tables S11–S13).

### Cause of death

Cause of death (COVID vs non-COVID) had a moderate to weak effect on end-of-life care outcomes (Tables S1–S7), with deaths due to COVID-19 associated with worse outcomes (Figure 1). In particular, participants bereaved due to COVID-19 were less likely to be involved in care decisions (*p* < 0.001) (Table S1) and less likely to be well supported by healthcare professionals immediately after the death (*p* < 0.001) (Table S4).

COVID-19 deaths were also associated with worse pandemic-related challenges, with weak to large effects sizes (Table 5; Figure 1). The total number of pandemic-related challenges was higher for COVID deaths compared to non-COVID (*p* < 0.001) (Table 5), and the odds of experiencing challenges were higher (Tables S8–S13; Figure 1). Significant increases in odds were seen for: unable to say goodbye as I would have liked (Table S10, OR = 0.348; 95% CI: 0.2–0.605); social isolation and loneliness (Table S12, OR = 0.439; 95% CI: 0.261–0.739); and limited contact with other close relatives/friends (Table S13, OR = 0.465; 95% CI: 0.254–0.852).

### Expectedness of the death

Whether the death was expected or not had a moderate to strong effect on four end-of-life care outcomes (Tables S1–S4), namely: an expected death led to the bereaved being more likely to be involved in care decisions, know the contact details for the professional responsible, receive information about the approaching death, and be well supported by healthcare professionals immediately after the death (all *p* < 0.001).

The bereaved person expecting their loved one to die was also significantly associated with fewer experiences of some pandemic-related challenges (often *p* < 0.001) (Table 5). Results of logistic regression supported these results, with a consistent picture of decreased odds for expected deaths, however these findings were not confirmed by the GLM (Tables S8–S13).

### Relationship to the deceased

Closer relationships (especially the deceased being a partner or child) compared with more distant relationships (distant family/colleague or friend) led to the bereaved person being more likely to know the contact details for the professional responsible for their loved one’s care (*p* = 0.001) and be provided with information about bereavement support services at the time of death (*p* < 0.001). Relationship to the deceased had a medium to large effect on these variables (Tables S1–S7).

Relationship to the deceased was also significantly associated with some pandemic-specific challenges. The inability to visit prior to death was highest if the deceased was a grandparent (77.8%) and lowest if the deceased was their child (33.3%) or partner (41.4%) compared with other groups (*p* < 0.001) (Table 5). Social isolation and loneliness was highest for bereaved partners (81.6%) compared with other groups (*p* < 0.001).

These results were confirmed in logistic regression and mixed-effects GLM. Relationships other than ‘partner’ had strongly increased odds of being unable to visit their friend or relative before death, for example, participants whose grandparent had died were nine times more likely not to have been able to visit them before death compared with bereaved partners (OR = 9.332, 95% CI: 2.033–42.841) (Table S8). Similar patterns occurred for limited contact in last days of life and being unable to say goodbye (Tables S9 and S10). Parents whose child had died had a decreased risk of limited contact with them in last days of their life compared with partners (GLM: OR = 0.094; 95% CI: 0.009–0.982) (Table S10). Odds of restricted funeral arrangements were reduced for all groups compared with the reference group of partner, although this was significant only for colleague or friend versus partner (OR = 0.233; 95% CI: 0.070–0.781) (Table S11). The odds of social isolation and loneliness were strongly and significantly reduced for all groups compared to the reference group of partner, for example, OR = 0.092 (95% CI: 0.028–0.297) for other family member (Table S12).

### Demographic characteristics

#### Qualifications

Participants with higher levels of qualification were significantly more likely to report being well supported by healthcare professionals immediately after the death (*p* = 0.028) and contacted by the hospital or care provider following the death (*p* = 0.011) (Tables S1–S8).

#### Deprivation and region of the UK

Decile of deprivation (England) and region of the UK occasionally had a strong or moderate effect on end-of-life care outcomes (Tables S1–S8). However, there were no significant differences (*p* > 0.05) and no obvious and consistent pattern across outcomes (region of the UK was therefore included in the mixed-effects GLM as a random-effect).

#### Gender

The effects of gender identity were generally weak (Tables S1–S7). Odds of social isolation and loneliness and separately limited contact with other close relatives/friends were higher for women compared with men, although this was only significant for limited contact with other close relatives/friends. The odds of restricted funeral arrangements were higher for women compared with men, although this was significant only for logistic regression (Table S13, GLM: OR = 2.496; 95% CI: 1.185–5.255).

#### Other demographic factors

Age of the deceased and the bereaved, religious belief, ethnicity, time since death, and same/different sex partnership had small effects, none of which were significant.

## Discussion

### Main findings

This study identified risk factors for poor experiences among people bereaved during the COVID-19 pandemic. We found that place, cause and expectedness of death and relationship with the deceased were associated with sub-optimal end-of-life care and challenging experiences in early bereavement. Place of death had a moderate to strong effect on end-of-life care outcomes, with deaths in hospitals and care homes in the UK associated with worse outcomes than deaths in a hospice or at home, reflecting pressures on health and social care during the pandemic. Odds of social isolation and loneliness in early bereavement were highest for hospital deaths compared with other settings. Deaths due to COVID-19 were moderately to weakly associated with worse experiences of end-of-life care and perceived professional support after the death. People bereaved by COVID-19 were also particularly impacted by pandemic-related challenges, such as being unable to visit their loved one, limited contact with other relatives/friends, and social isolation and loneliness. This could reflect quarantining requirements, but also lockdown restrictions (first introduced in the UK on 23 March 2020), fear and anxiety around catching/spreading the virus, and the social challenges and alienation associated with COVID-19 bereavement.^12,24,25^

When a death was unexpected the bereaved was less likely to be involved in care, feel supported by healthcare professionals or be contacted afterwards, and the odds of social isolation and loneliness were higher. Partners and parents of the deceased were more likely to report supportive end-of-life care compared with distant family members/friends, however social isolation and loneliness was highest among partners.

People with higher levels of qualification reported better support from healthcare professionals and more contact following the death, possibly because people with higher levels of education are more able to elicit professionals’ support. Compared to men, women seemed more likely to report limited contact with other close relatives or friends. Both of these findings warrant further investigation.

### What this study adds

Bereaved people reported worse experiences of hospital and care home deaths than deaths at home or hospice, as in pre-pandemic studies.^26–29^ Modelling of routine data from the first 10 weeks of the pandemic in the UK (7 March–15 May 2020), found deaths in care homes increased by 220%, and home and hospital deaths by 77% and 90% respectively, while hospice deaths fell by 20%.^30^ The increase in home deaths was sustained^31^ and hospices shifted their resources to the community.^32^ Our findings suggest that despite the rise in home deaths during the pandemic, they were associated with better experiences of end-of-life care than deaths in other settings, indicating that primary and community care services were comparatively successful in supporting home deaths, despite the additional pressures on services.^33^

Our findings could help explain why the pandemic may increase levels of prolonged grief disorder and other longer-term poor bereavement outcomes^34–36^ For example, among the COVID-19 bereaved, poorer bereavement outcomes might be explained by the higher likelihood of poor end-of-life care experiences as well as the increased likelihood of pandemic-related challenges due to infection control restrictions. The study’s longitudinal and qualitative data will throw further light on such outcomes and experiences in this sample.

We found increased levels of social isolation and loneliness among people bereaved due to COVID-19, with partners at particular risk. In contrast, in a survey in the Netherlands^34^ satisfaction with social support did not differ between people bereaved by COVID-19 versus other types of deaths – however, given the small number of COVID-19 deaths (*n* = 49) these findings should be treated with caution. A US survey of people bereaved by COVID-19 (*n* = 307) found a close relationship with the deceased was associated with reduced functional impairment due to the loss.^35^ In China (*n* = 422), the death of a close relation due to COVID-19 was associated with more severe grief symptoms.^37^ The higher levels of loneliness and social isolation we observed amongst bereaved partners may help explain these associations.

### Strengths and weaknesses

The study sample was large, with good spread across geographical areas, education and deprivation, but was biased towards female and white respondents, despite targeted advertising to men and people from ethnic minority communities. By recruiting mostly online, we were less likely to reach the very old or other digitally marginalised groups, hence the high levels of social isolation we identified might under-estimate levels in the general bereaved population. Convenience sampling might have resulted in more people with negative experiences completing the survey. Despite these limitations, group sizes were sufficient to enable comparisons (although not to the level of specific ethnic groups) and, while not providing population-level prevalence data, the sample does enable identification of risk factors to inform future practice and policy.

Another strength of this study is the use of an explicit and structured statistical analysis plan. A Directed Acyclic Graph was used to visualise the relationships between variables and appropriate hypotheses were thereby generated (e.g. COVID-19 are deaths associated with higher levels of pandemic-specific end-of-life and social issues than non-COVID deaths). These hypotheses were tested for statistically by using both univariate calculations and mixed-effects logistic regression (i.e. the GLM).

### Implications

The evidence of sub-optimal end-of-life care demonstrates the difficulty of adequately supporting families during the pandemic, but also highlights areas for improvement. Communication with relatives must be prioritised and contact between loved ones at the end of life facilitated and optimised, even in the context of a pandemic. It is therefore crucial that end-of-life care providers are prioritised when supplies of personal protective equipment are overstretched, so that they are able to offer in-person visits, however there is evidence that this did not occur in 2020.^38^ Patients admitted to hospital with COVID-19 often experience an unpredictable clinical course with high risk of sudden death. Discussions with next of kin should happen early, with this risk explained clearly and compassionately. Partners bereaved due to an unexpected COVID-19 death in hospital may be at particular risk of poor outcomes and need additional follow-up and support, particularly when they live alone or have a previous history of mental disorders.^39^ However, many challenges were experienced by people bereaved by non-COVID-19 deaths, and difficulties across the bereaved population should not be minimised. In particular, after sudden deaths of any cause bereaved people require attention and follow-up, given perceptions of poor support after death. Clear, consistent national guidance on hospital, hospice and care home visiting is essential to ensure equity and support staff. We found only a third of bereaved people had been given information about bereavement support services. Signposting at the time of death and in follow-up must be improved to ensure people know how and where to seek professional support and help alleviate access barriers.^40^

Further research is needed to examine the impact of end-of-life care experiences and pandemic-related social challenges on bereavement outcomes, and to determine the prevalence of poor bereavement outcomes including prolonged grief disorder among people bereaved during the pandemic, with comparisons to non-pandemic times. This is crucial given discrepancies in emerging evidence.^41–43^ The experiences of bereaved men and people from Black and minority ethnic communities during the pandemic require further investigation.

## Conclusions

Place, cause and expectedness of deaths and relationship to the deceased were risk factors for sub-optimal end-of-life care and challenging experiences in early bereavement during the pandemic. People bereaved by COVID-19 and partners bereaved by all causes of death were at particular risk of social isolation and loneliness. To learn from COVID-19 as a mass bereavement event, these findings should inform optimal clinical practice, bereavement support and the policy response.

## Supplemental Material

sj-pdf-1-pmj-10.1177_02692163221074876 – Supplemental material for Risk factors associated with poorer experiences of end-of-life care and challenges in early bereavement: Results of a national online survey of people bereaved during the COVID-19 pandemic Supplemental material, sj-pdf-1-pmj-10.1177_02692163221074876 for Risk factors associated with poorer experiences of end-of-life care and challenges in early bereavement: Results of a national online survey of people bereaved during the COVID-19 pandemic by Lucy Ellen Selman, DJJ Farnell, M Longo, S Goss, K Seddon, A Torrens-Burton, CR Mayland, D Wakefield, B Johnston, A Byrne and E Harrop in Palliative Medicine

sj-pdf-2-pmj-10.1177_02692163221074876 – Supplemental material for Risk factors associated with poorer experiences of end-of-life care and challenges in early bereavement: Results of a national online survey of people bereaved during the COVID-19 pandemic Supplemental material, sj-pdf-2-pmj-10.1177_02692163221074876 for Risk factors associated with poorer experiences of end-of-life care and challenges in early bereavement: Results of a national online survey of people bereaved during the COVID-19 pandemic by Lucy Ellen Selman, DJJ Farnell, M Longo, S Goss, K Seddon, A Torrens-Burton, CR Mayland, D Wakefield, B Johnston, A Byrne and E Harrop in Palliative Medicine

sj-pdf-3-pmj-10.1177_02692163221074876 – Supplemental material for Risk factors associated with poorer experiences of end-of-life care and challenges in early bereavement: Results of a national online survey of people bereaved during the COVID-19 pandemic Supplemental material, sj-pdf-3-pmj-10.1177_02692163221074876 for Risk factors associated with poorer experiences of end-of-life care and challenges in early bereavement: Results of a national online survey of people bereaved during the COVID-19 pandemic by Lucy Ellen Selman, DJJ Farnell, M Longo, S Goss, K Seddon, A Torrens-Burton, CR Mayland, D Wakefield, B Johnston, A Byrne and E Harrop in Palliative Medicine
