# Supplementary material for: Risk factors associated with poorer experiences of end-of-life care and challenges in early bereavement: Results of a national online survey of people bereaved during the COVID-19 pandemic
Source: Palliat Med. 2022 Feb 17;36(4):717–29. doi: 10.1177/02692163221074876 (PMC9005832; doi:10.1177/02692163221074876)
Supplement: sj-pdf-2-pmj-10.1177_02692163221074876 – Supplemental material for Risk factors associated with poorer experiences of end-of-life care and challenges in early bereavement: Results of a national online survey of people bereaved during the COVID-19 pandemic [file sj-pdf-2-pmj-10.1177_02692163221074876.pdf]

Supplementary file 2: Survey advertisement

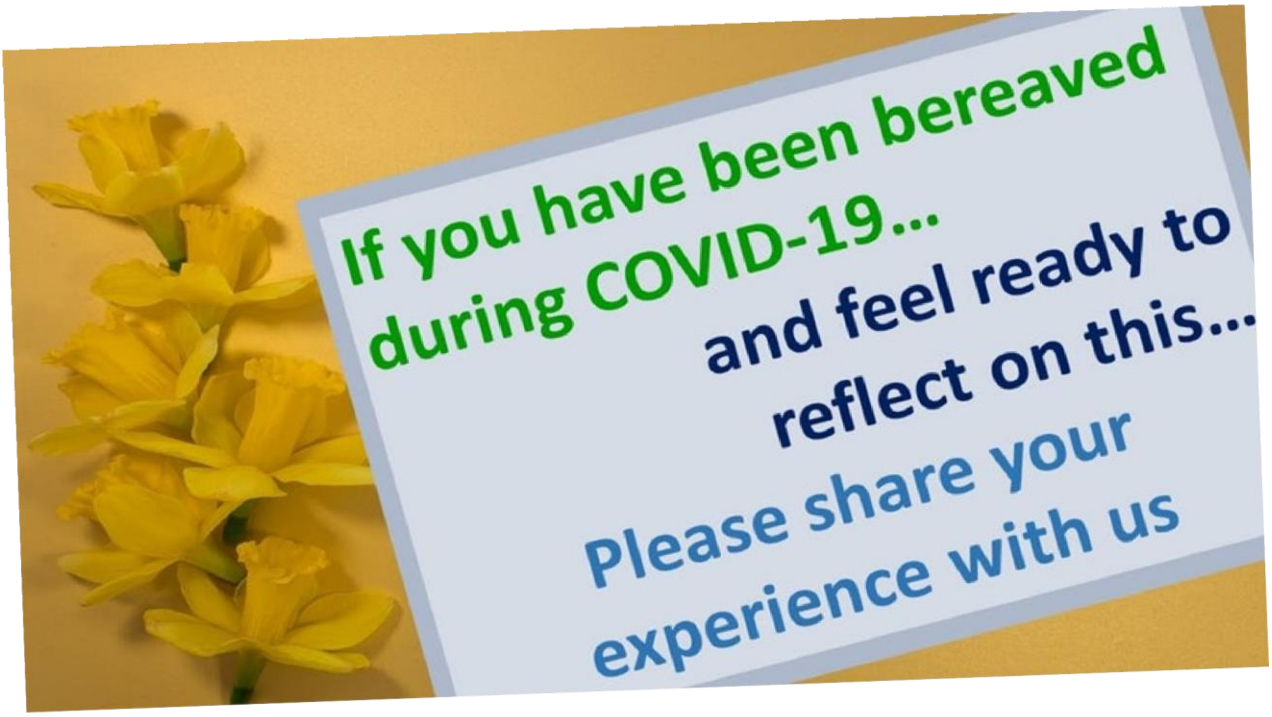


Cardiff University and the University of Bristol are conducting a **survey** looking at the **grief experiences** and **support needs** of people **bereaved** during the **pandemic.**

By conducting this survey we hope to identify ways of improving the care provided at the end of life and during bereavement.

If you have lost a loved one to COVID-19 or another cause of death during the pandemic, and would like to share your experience in our survey, please **click here** or visit www.covidbereavement.com

Or contact Emily Harrop: **harrope@cardiff.ac.uk**, **tel: 02920 687184** for further information or a paper copy of the survey.

## Thank you!

Cardiff University
Prifysgol Caerdydd
E·S·R·C Economic & Social Research Council
University of Bristol
Marie Curie
Care and support through terminal illness
Canolfan Ymchwil Gofal Lliniarol Marie Curie
Palliative Care Research Centre
Cardiff Caerdydd
