# Supplementary material for: Risk factors associated with poorer experiences of end-of-life care and challenges in early bereavement: Results of a national online survey of people bereaved during the COVID-19 pandemic
Source: Palliat Med. 2022 Feb 17;36(4):717–29. doi: 10.1177/02692163221074876 (PMC9005832; doi:10.1177/02692163221074876)
Supplement: sj-pdf-3-pmj-10.1177_02692163221074876 – Supplemental material for Risk factors associated with poorer experiences of end-of-life care and challenges in early bereavement: Results of a national online survey of people bereaved during the COVID-19 pandemic [file sj-pdf-3-pmj-10.1177_02692163221074876.pdf]

Supplementary tables

		n	Never	Sometimes	Usually	Always	Mean	Median	SD	*h* (Effect Size), *P*
Highest qualification	None / GCSEs	96	37.5%	24.0%	12.5%	26.0%	2.27	2.39	2.51	0.29 (Small) 0.183
	A-level etc.	115	27.8%	28.7%	20.0%	23.5%	2.00	2.00	2.00	
	HND / Degree etc.	356	23.9%	29.8%	17.7%	28.7%	1.22	1.13	1.14	
Where did they die?	Hospital	350	35.4%	29.1%	15.4%	20.0%	2.20	2.00	1.13	0.88 (Large) <0.001*
	Home	100	13.0%	24.0%	19.0%	44.0%	2.94	3.00	1.10	
	Hospice	35	8.6%	22.9%	22.9%	45.7%	3.06	3.00	1.03	
	Care home	78	17.9%	34.6%	19.2%	28.2%	2.58	2.00	1.09	
	Other / Don't Know	5	20.0%	20.0%	0.0%	60.0%	3.00	4.00	1.41	
Cause of death	COVID (Confirmed or Suspected)	266	39.1%	29.7%	13.9%	17.3%	2.09	2.00	1.10	0.47 (Medium) <0.001*
	Non-Covid	303	16.8%	27.4%	20.1%	35.6%	2.75	3.00	1.12	
Gender Identity	Male	60	18.3%	30.0%	21.7%	30.0%	2.63	3.00	1.10	0.1 (Small) 0.089
	Female	506	28.3%	27.9%	16.8%	27.1%	2.43	2.00	1.16	
	Other	3	0.0%	100.0%	0.0%	0.0%	2.45	2.00	1.16	
Ethnicity	Non-BAME	541	26.8%	28.7%	17.2%	27.4%	2.41	2.00	1.19	0.06 (Small) 0.979
	BAME	27	29.6%	25.9%	18.5%	25.9%	2.53	2.00	1.18	
Who was it that died?	Partner	140	25.7%	27.1%	15.7%	31.4%	2.53	2.00	1.18	0.60 (Medium) 0.154
	Parent	356	25.6%	29.5%	17.7%	27.2%	2.47	2.00	1.14	
	Grandparent	25	32.0%	28.0%	16.0%	24.0%	2.32	2.00	1.18	
	Sibling	15	46.7%	20.0%	20.0%	13.3%	2.00	2.00	1.13	
	Child	9	22.2%	11.1%	33.3%	33.3%	2.78	3.00	1.20	
	Other family member	20	30.0%	40.0%	15.0%	15.0%	2.15	2.00	1.04	
	Colleague or friend	5	–	–	–	–				
Religious beliefs	Yes	280	26.4%	25.7%	17.9%	30.0%	2.51	2.00	1.18	0.11 (Small) 0.463
	No	239	26.4%	31.0%	18.0%	24.7%	2.41	2.00	1.13	
Was the death expected?	Yes	94	5.3%	22.3%	26.6%	45.7%	3.13	3.00	0.94	0.56 (Medium) <0.001*
	No	439	32.6%	30.1%	15.0%	22.3%	2.27	2.00	1.14	

Region of the UK	Northern Ireland	20	20.0%	25.0%	10.0%	45.0%	2.8	3	1.24	0.69 (Medium / Large), 0.268
	Wales	50	22.0%	34.0%	16.0%	28.0%	2.5	2	1.129	
	West Midlands	45	33.3%	31.1%	11.1%	24.4%	2.27	2	1.176	
	North East	30	26.7%	26.7%	10.0%	36.7%	2.57	2	1.251	
	Greater London	49	24.5%	18.4%	26.5%	30.6%	2.63	3	1.167	
	South East	62	30.6%	27.4%	27.4%	14.5%	2.26	2	1.055	
	North West	80	25.0%	31.3%	11.3%	32.5%	2.51	2	1.191	
	East Midlands	35	40.0%	25.7%	14.3%	20.0%	2.14	2	1.167	
	South West	42	19.0%	31.0%	16.7%	33.3%	2.64	2.5	1.144	
	Scotland	41	22.0%	26.8%	12.2%	39.0%	2.68	3	1.213	
	East of England	33	27.3%	33.3%	27.3%	12.1%	2.24	2	1.001	
	Yorkshire & the Humber	41	22.0%	31.7%	12.2%	34.1%	2.59	2	1.183	
IMD Decile (England only)	1	23	43.5%	21.7%	8.7%	26.1%	2.17	2	1.267	0.50 (Medium), 0.552
	2	35	25.7%	25.7%	14.3%	34.3%	2.57	2	1.22	
	3	41	26.8%	24.4%	12.2%	36.6%	2.59	2	1.245	
	4	45	20.0%	40.0%	15.6%	24.4%	2.44	2	1.078	
	5	50	24.0%	30.0%	12.0%	34.0%	2.56	2	1.198	
	6	43	30.2%	25.6%	11.6%	32.6%	2.47	2	1.241	
	7	47	25.5%	31.9%	17.0%	25.5%	2.43	2	1.137	
	8	43	25.6%	25.6%	30.2%	18.6%	2.42	2	1.074	
	9	36	27.8%	25.0%	25.0%	22.2%	2.42	2	1.131	
	10	43	27.9%	32.6%	25.6%	14.0%	2.26	2	1.131	

* $P < 0.05$

Table S1: Descriptive statistics (row percentages, means, and medians) for question "Did the care professionals involve you in decisions about the care for your sick loved one?". *P*-values are from chi-squared analysis of a contingency table. Effect sizes are estimated from Cohen's h, where $h = 0.2$: small effect, $h = 0.5$: medium effect, $h \geq 0.8$: large effect.

		n	Yes	No	*h* (Effect Size), *P*
Highest qualification	None / GCSEs	85	56.5%	43.5%	0.30 (Small), 0.186
	A-level etc.	107	68.2%	31.8%	
	HND / Degree etc.	352	65.9%	34.1%	
Where did they die?	Hospital	329	55.0%	45.0%	0.80 (Large), 0.001*
	Home	101	80.2%	19.8%	
	Hospice	34	70.6%	29.4%	
	Care home	77	83.1%	16.9%	
	Other / Don't Know	5	60.0%	40.0%	
Cause of death	COVID (Confirmed or Suspected)	255	58.4%	41.6%	0.31 (Small), 0.005*
	Non-Covid	291	70.1%	29.9%	
Gender Identity	Male	58	67.2%	32.8%	0.07 (Small), 0.925
	Female	482	64.5%	35.5%	
	Other	4	75.0%	25.0%	
Ethnicity	Non-BAME	519	64.4%	35.6%	0.36 (Small / Medium), 0.213
	BAME	26	76.9%	23.1%	
Who was it that died?	Partners	127	74.0%	26.0%	1.11 (Large), 0.001*
	Parents	335	65.1%	34.9%	
	Grandparents	27	63.0%	37.0%	
	Sibling	16	31.3%	68.8%	
	Child	8	62.5%	37.5%	
	Other family member	23	52.2%	47.8%	
	Colleague or friend	11	27.3%	72.7%	
Religious beliefs	Yes	266	63.9%	36.1%	0.14 (Small), 0.253
	No	229	69.0%	31.0%	
Was the death expected?	Yes	94	86.2%	13.8%	0.83 (Large), <0.001*
	No	417	58.5%	41.5%	

Region of the UK	Northern Ireland	20	65.0%	35.0%	0.52 (Medium), 0.778
	Wales	50	60.0%	40.0%	
	West Midlands	41	61.0%	39.0%	
	North East	27	77.8%	22.2%	
	Greater London	52	63.5%	36.5%	
	South East	52	63.5%	36.5%	
	North West	82	73.2%	26.8%	
	East Midlands	34	58.8%	41.2%	
	South West	41	73.2%	26.8%	
	Scotland	40	70.0%	30.0%	
	East of England	32	62.5%	37.5%	
	Yorkshire & the Humber	38	65.8%	34.2%	
IMD Decile (England only)	1	23	65.2%	34.8%	0.56 (Medium), 0.861
	2	34	70.6%	29.4%	
	3	37	67.6%	32.4%	
	4	45	64.4%	35.6%	
	5	50	66.0%	34.0%	
	6	39	74.4%	25.6%	
	7	45	66.7%	33.3%	
	8	48	66.7%	33.3%	
	9	32	53.1%	46.9%	
	10	37	73.0%	27.0%	

* $P < 0.05$

Table S2: Descriptive statistics (row percentages) for the question “Did you know the contact details for the professional responsible for their care?”. *P*-values are from chi-squared analysis of a contingency table. Effect sizes are estimated from Cohen’s h, where $h = 0.2$: small effect, $h = 0.5$: medium effect, $h \geq 0.8$: large effect.

		n	No, not at all (=1)	A bit of information (=2)	Yes, fully informed (=3)	Mean	Median	SD	*h* (Effect Size), *P*
Highest qualification	None / GCSEs	97	26.8%	40.2%	33.0%	2.06	2.00	0.78	0.25 (Small), 0.113
	A-level etc.	123	17.9%	51.2%	30.9%	2.13	2.00	0.69	
	HND / Degree etc.	404	19.1%	41.3%	39.6%	2.21	2.00	0.74	
Where did they die?	Hospital	387	20.9%	44.7%	34.4%	2.13	2.00	0.73	0.59 (Medium), 0.22
	Home	112	22.3%	42.0%	35.7%	2.13	2.00	0.75	
	Hospice	36	11.1%	30.6%	58.3%	2.47	3.00	0.70	
	Care home	84	16.7%	44.0%	39.3%	2.23	2.00	0.72	
	Other / Don't Know	5	20.0%	20.0%	60.0%	2.40	3.00	0.89	
Cause of death	COVID (Confirmed or Suspected)	295	19.0%	48.8%	32.2%	2.13	2.00	0.70	0.24 (Small), 0.018*
	Non-Covid	330	21.2%	37.9%	40.9%	2.20	2.00	0.76	
Gender Identity	Male	69	18.8%	34.8%	46.4%	2.28	2.00	0.77	0.24 (Small), 0.072
	Female	551	20.0%	44.5%	35.6%	2.16	2.00	0.73	
	Other	5	60.0%	20.0%	20.0%	1.60	1.00	0.89	
Ethnicity	Non-BAME	597	19.8%	43.6%	36.7%	2.17	2.00	0.73	0.18 (Small), 0.501
	BAME	28	28.6%	35.7%	35.7%	2.07	2.00	0.81	
Who was it that died?	Partners	132	18.9%	36.4%	44.7%	2.26	2.00	0.76	0.95 (Large), 0.096
	Parents	375	18.4%	45.3%	36.3%	2.18	2.00	0.72	
	Grandparent	43	25.6%	44.2%	30.2%	2.05	2.00	0.75	
	Sibling	21	19.0%	38.1%	42.9%	2.24	2.00	0.77	
	Child	8	12.5%	75.0%	12.5%	2.00	2.00	0.54	
	Other family member	32	28.1%	46.9%	25.0%	1.97	2.00	0.74	
	Colleague or friend	15	46.7%	26.7%	26.7%	1.80	2.00	0.86	
Religious beliefs	Yes	302	18.5%	41.1%	40.4%	2.22	2.00	0.74	0.13 (Small), 0.284
	No	265	22.3%	43.4%	34.3%	2.12	2.00	0.74	
Was the death expected?	Yes	106	12.3%	32.1%	55.7%	2.43	3.00	0.70	0.54 (Medium), <0.001*
	No	478	22.4%	46.0%	31.6%	2.09	2.00	0.73	

Region of the UK	Northern Ireland	22	22.7%	27.3%	50.0%	2.27	2.5	0.827	0.67 (Medium / Large), 0.299
	Wales	57	28.1%	49.1%	22.8%	1.95	2	0.718	
	West Midlands	47	14.9%	44.7%	40.4%	2.26	2	0.706	
	North East	33	18.2%	54.5%	27.3%	2.09	2	0.678	
	Greater London	63	25.4%	34.9%	39.7%	2.14	2	0.8	
	South East	70	17.1%	52.9%	30.0%	2.13	2	0.679	
	North West	88	18.2%	44.3%	37.5%	2.19	2	0.725	
	East Midlands	36	16.7%	47.2%	36.1%	2.19	2	0.71	
	South West	43	14.0%	32.6%	53.5%	2.4	3	0.728	
	Scotland	42	21.4%	31.0%	47.6%	2.26	2	0.798	
	East of England	35	25.7%	40.0%	34.3%	2.09	2	0.781	
	Yorkshire & the Humber	46	15.2%	45.7%	39.1%	2.24	2	0.705	
IMD Decile (England only)	1	26	15.4%	46.2%	38.5%	2.23	2	0.71	0.54 (Medium), 0.169
	2	40	30.0%	27.5%	42.5%	2.13	2	0.853	
	3	45	8.9%	51.1%	40.0%	2.31	2	0.633	
	4	50	16.0%	52.0%	32.0%	2.16	2	0.681	
	5	58	25.9%	32.8%	41.4%	2.16	2	0.812	
	6	44	15.9%	45.5%	38.6%	2.23	2	0.711	
	7	49	14.3%	49.0%	36.7%	2.22	2	0.685	
	8	51	9.8%	45.1%	45.1%	2.35	2	0.658	
	9	40	32.5%	37.5%	30.0%	1.98	2	0.8	
	10	47	17.0%	51.1%	31.9%	2.15	2	0.691	

* $P < 0.05$

Table S3: Descriptive statistics (row percentages, means, and medians) for the question "Did you receive information about the approaching death?". *P*-values are from chi-squared analysis of a contingency table. Effect sizes are estimated from Cohen's h, where $h = 0.2$: small effect, $h = 0.5$: medium effect, $h \geq 0.8$: large effect.

		n	Very well supported	Fairly well supported	A little bit supported	Not at all supported	Mean	Median	SD	*h* (Effect Size), *P*
Highest qualification	None / GCSEs	95	9.5%	9.5%	27.4%	53.7%	3.25	4.00	0.98	0.34 (Small /Medium), 0.028*
	A-level etc.	122	18.0%	15.6%	20.5%	45.9%	2.94	3.00	1.16	
	HND / Degree etc.	371	17.3%	20.5%	23.5%	38.8%	2.84	3.00	1.12	
Where did they die?	Hospital	358	15.1%	17.3%	23.2%	44.4%	2.97	3.00	1.11	0.93 (Large), 0.003*
	Home	113	23.0%	16.8%	26.5%	33.6%	2.71	3.00	1.16	
	Hospice	33	30.3%	30.3%	24.2%	15.2%	2.24	2.00	1.06	
	Care home	78	5.1%	16.7%	20.5%	57.7%	3.31	4.00	0.93	
	Other / Don't Know	7	14.3%	14.3%	28.6%	42.9%	3.00	3.00	1.16	
Cause of death	COVID (Confirmed or Suspected)	269	9.3%	12.3%	24.9%	53.5%	3.23	4.00	0.99	0.45 (Medium), <0.001*
	Non-Covid	321	21.8%	22.4%	22.4%	33.3%	2.67	3.00	1.15	
Gender Identity	Male	61	16.4%	23.0%	23.0%	37.7%	2.82	3.00	1.12	0.06 (Small), 0.892
	Female	526	16.0%	17.1%	23.6%	43.3%	2.94	3.00	1.12	
	Other	3	0.0%	33.3%	33.3%	33.3%	3.00	3.00	1.00	
Ethnicity	Non-BAME	561	15.7%	18.4%	23.5%	42.4%	2.93	3.00	1.11	Weak, 0.476
	BAME	28	21.4%	7.1%	25.0%	46.4%	2.96	3.00	1.20	
Who was it that died?	Partners	145	21.4%	19.3%	22.8%	36.6%	2.98	3.00	1.11	0.43 (Medium), 0.494
	Parents	369	15.4%	15.7%	23.8%	45.0%	3.00	3.00	0.98	
	Grandparents	24	4.2%	33.3%	20.8%	41.7%	3.27	3.00	0.80	
	Sibling	15	0.0%	20.0%	33.3%	46.7%	2.92	3.00	1.17	
	Child	12	16.7%	16.7%	25.0%	41.7%	2.87	3.00	1.10	
	Other family member	23	13.0%	26.1%	21.7%	39.1%	3.00	4.00	1.73	
	Colleague or friend	3	–	–	–	–				
Religious beliefs	Yes	286	18.9%	18.2%	19.9%	43.0%	2.87	3.00	1.16	0.19 (Small), 0.038*
	No	252	12.3%	16.7%	29.0%	42.1%	3.01	3.00	1.04	
	Yes	94	28.7%	20.2%	28.7%	22.3%	2.45	3.00	1.13	

Was the death expected?	No	458	12.7%	16.8%	22.9%	47.6%	3.05	3.00	1.07	0.54 (Medium), <0.001*
Region of the UK	Northern Ireland	19	26.3%	10.5%	26.3%	36.8%	2.74	3	1.24	0.66 (Medium / Large), 0.034*
	Wales	50	8.0%	14.0%	28.0%	50.0%	3.2	3.5	0.969	
	West Midlands	48	16.7%	16.7%	8.3%	58.3%	3.08	4	1.2	
	North East	31	9.7%	35.5%	25.8%	29.0%	2.74	3	0.999	
	Greater London	51	23.5%	19.6%	25.5%	31.4%	2.65	3	1.163	
	South East	62	14.5%	12.9%	21.0%	51.6%	3.1	4	1.112	
	North West	84	17.9%	17.9%	25.0%	39.3%	2.86	3	1.132	
	East Midlands	34	17.6%	11.8%	32.4%	38.2%	2.91	3	1.111	
	South West	46	19.6%	17.4%	21.7%	41.3%	2.85	3	1.173	
	Scotland	45	26.7%	28.9%	13.3%	31.1%	2.49	2	1.199	
	East of England	35	2.9%	8.6%	31.4%	57.1%	3.43	4	0.778	
	Yorkshire & the Humber	43	14.0%	14.0%	32.6%	39.5%	2.98	3	1.058	
IMD Decile (England only)	1	24	12.5%	20.8%	16.7%	50.0%	3.04	3.5	1.122	0.47 (Medium), 0.941
	2	39	17.9%	17.9%	25.6%	38.5%	2.85	3	1.136	
	3	42	16.7%	14.3%	38.1%	31.0%	2.83	3	1.057	
	4	45	11.1%	17.8%	26.7%	44.4%	3.04	3	1.043	
	5	51	21.6%	17.6%	21.6%	39.2%	2.78	3	1.189	
	6	44	9.1%	15.9%	22.7%	52.3%	3.18	4	1.018	
	7	50	20.0%	20.0%	18.0%	42.0%	2.82	3	1.19	
	8	45	22.2%	17.8%	22.2%	37.8%	2.76	3	1.19	
	9	39	12.8%	15.4%	30.8%	41.0%	3	3	1.051	
	10	42	11.9%	16.7%	19.0%	52.4%	3.12	4	1.087	

* P < 0.05

Table S4: Descriptive statistics (row percentages, means, and medians) for the question “Did you feel well supported by the healthcare professionals immediately after the death of your loved one?”. *P*-values are from chi-squared analysis of a contingency table. Effect sizes are estimated from Cohen’s h, where $h = 0.2$: small effect, $h = 0.5$: medium effect, $h \geq 0.8$: large effect.

		n	Yes	No	*h* (Effect Size), *P*
Highest qualification	None / GCSEs	93	31.2%	68.8%	0.43 (Medium), 0.011*
	A-level etc.	117	41.0%	59.0%	
	HND / Degree etc.	360	48.1%	51.9%	
Where did they die?	Hospital	359	42.3%	57.7%	0.51 (Medium), 0.216
	Home	95	44.2%	55.8%	
	Hospice	33	63.6%	36.4%	
	Care home	76	42.1%	57.9%	
	Other / Don’t Know	8	50.0%	50.0%	
Cause of death	COVID (Confirmed or Suspected)	269	40.9%	59.1%	0.13 (Small), 0.206
	Non-Covid	303	46.2%	53.8%	
Gender Identity	Male	60	43.3%	56.7%	0.01 (None / Small), 0.614
	Female	510	43.9%	56.1%	
	Other	2	0.0%	100.0%	
Ethnicity	Non-BAME	543	44.4%	55.6%	0.31 (Small), 0.243
	BAME	28	32.1%	67.9%	
Who was it that died?	Partners	142	52.8%	47.2%	0.48 (Medium), 0.353
	Parents	355	41.1%	58.9%	
	Grandparent	24	37.5%	62.5%	
	Sibling	13	38.5%	61.5%	
	Child	11	45.5%	54.5%	
	Other family member	22	40.9%	59.1%	
	Colleague or friend	6	33.3%	66.7%	
Religious beliefs	Yes	280	45.4%	54.6%	0.07 (None / Small), 0.537
	No	243	42.4%	57.6%	
Was the death expected?	Yes	88	56.8%	43.2%	0.36 (Small /

	No	45<br>1	41.2<br>%	58.8%	Medium), 0.009*
Region of the UK	Northern Ireland	19	68.4<br>%	31.6%	0.90 (Large), 0.36
	Wales	49	46.9<br>%	53.1%	
	West Midlands	48	37.5<br>%	62.5%	
	North East	30	50.0<br>%	50.0%	
	Greater London	52	44.2<br>%	55.8%	
	South East	61	44.3<br>%	55.7%	
	North West	83	47.0<br>%	53.0%	
	East Midlands	33	39.4<br>%	60.6%	
	South West	41	39.0<br>%	61.0%	
	Scotland	45	55.6<br>%	44.4%	
	East of England	33	30.3<br>%	69.7%	
	Yorkshire & the Humber	42	40.5<br>%	59.5%	
IMD Decile (England only)	1	23	30.4<br>%	69.6%	0.64 (Medium / Large), 0.488
	2	39	46.2<br>%	53.8%	
	3	41	46.3<br>%	53.7%	
	4	44	31.8<br>%	68.2%	
	5	52	44.2<br>%	55.8%	
	6	42	42.9<br>%	57.1%	
	7	48	45.8<br>%	54.2%	
	8	44	34.1<br>%	65.9%	
	9	37	56.8<br>%	43.2%	
	10	41	39.0<br>%	61.0%	

* P < 0.05

Table S5: Descriptive statistics (row percentages) for the question “Were you contacted again by the hospital or care provider following their death?”. *P*-values are from chi-squared analysis of a contingency table. Effect sizes are estimated from Cohen’s *h*, where h = 0.2: small effect, h = 0.5: medium effect, $h \geq 0.8$: large effect.

		n	Yes	No	*h* (Effect Size), *P*
Highest qualification	None / GCSEs	108	21.3%	78.7%	0.08 (Small) 0.848
	A-level etc.	132	19.7%	80.3%	
	HND / Degree etc.	468	22.0%	78.0%	
Where did they die?	Hospital	410	25.9%	74.1%	1.47 (Large), <0.001*
	Home	158	15.2%	84.8%	
	Hospice	37	48.6%	51.4%	
	Care home	91	4.4%	95.6%	
	Other / don't know	13	7.7%	92.3%	
Cause of death	COVID (Confirmed or Suspected)	311	17.7%	82.3%	0.22 (Small), 0.028*
	Non-Covid	399	24.6%	75.4%	
Gender Identity	Male	74	27.0%	73.0%	0.19 (Small), 0.529
	Female	628	21.0%	79.0%	
	Other	7	14.3%	85.7%	
Ethnicity	Non-BAME	676	21.9%	78.1%	0.23 (Small), 0.399
	BAME	33	15.2%	84.8%	
Who was it that died?	Partners	152	34.9%	65.1%	1.17 (Large), <0.001*
	Parents	395	20.8%	79.2%	
	Grandparent	54	9.3%	90.7%	
	Sibling	23	8.7%	91.3%	
	Child	15	33.3%	66.7%	
	Other family member	46	10.9%	89.1%	
	Colleague or friend	26	3.8%	96.2%	
Religious beliefs	Yes	340	21.5%	78.5%	0.03 (None / Small), 0.849
	No	301	22.3%	77.7%	
Was the death expected?	Yes	113	24.8%	75.2%	0.12 (Small), 0.382
	No	552	21.0%	79.0%	
Region of the UK	Northern Ireland	26	15.4%	84.6%	0.64 (Medium / Large), 0.283
	Wales	63	14.3%	85.7%	
	West Midlands	52	17.3%	82.7%	
	North East	40	12.5%	87.5%	
	Greater London	68	27.9%	72.1%	
	South East	78	21.8%	78.2%	
	North West	95	24.2%	75.8%	
	East Midlands	39	30.8%	69.2%	
	South West	51	17.6%	82.4%	
	Scotland	53	32.1%	67.9%	
	East of England	39	25.6%	74.4%	
	Yorkshire & the Humber	55	20.0%	80.0%	
IMD Decile (England only)	1	26	34.6%	65.4%	0.50 (Medium), 0.805
	2	45	24.4%	75.6%	
	3	49	20.4%	79.6%	

	4	52	26.9%	73.1%	
	5	64	21.9%	78.1%	
	6	52	28.8%	71.2%	
	7	58	20.7%	79.3%	
	8	57	19.3%	80.7%	
	9	46	19.6%	80.4%	
	10	50	18.0%	82.0%	

* P < 0.05

Table S6: Descriptive statistics for the question "Did they provide information about bereavement support services? – Yes (at the time of death)". *P*-values are from chi-squared analysis of a contingency table. Effect sizes are estimated from Cohen's *h*, where $h = 0.2$: small effect, $h = 0.5$: medium effect, $h \geq 0.8$: large effect.

		n	Yes	No	*h* (Effect Size), *P*
Highest qualification	None / GCSEs	108	17.6%	82.4%	0.27 (Small), 0.09
	A-level etc.	132	21.2%	78.8%	
	HND / Degree etc.	468	13.7%	86.3%	
Where did they die?	Hospital	410	17.8%	82.2%	1.27 (Large), <0.001*
	Home	158	11.4%	88.6%	
	Hospice	37	40.5%	59.5%	
	Care home	91	4.4%	95.6%	
	Other / DON'T KNOW	13	7.7%	92.3%	
Cause of death	COVID (Confirmed or Suspected)	311	16.7%	83.3%	0.07 (None / Small), 0.532
	Non-Covid	399	14.8%	85.2%	
Gender Identity	Male	74	16.2%	83.8%	0.02 (None), 0.615
	Female	628	15.8%	84.2%	
	Other	7	0.0%	100.0%	
Ethnicity	Non-BAME	676	15.8%	84.2%	0.15 (Small), 0.638
	BAME	33	12.1%	87.9%	
Who was it that died?	Partners	152	23.7%	76.3%	1.50 (Large), 0.09
	Parents	395	15.2%	84.8%	
	Grandparents	54	5.6%	94.4%	
	Sibling	23	21.7%	78.3%	
	Child	15	26.7%	73.3%	
	Other family member	46	6.5%	93.5%	
	Colleague or friend	26	0.0%	100.0%	
Religious beliefs	Yes	340	18.2%	81.8%	0.18 (Small), 0.104
	No	301	13.3%	86.7%	
Was the death expected?	Yes	113	19.5%	80.5%	0.15 (Small), 0.323
	No	552	15.2%	84.8%	
Region of the UK	Northern Ireland	26	19.2%	80.8%	

	Wales	63	7.9%	92.1%	0.59 (Medium), 0.342
	West Midlands	52	19.2 %	80.8%	
	North East	40	17.5 %	82.5%	
	Greater London	68	14.7 %	85.3%	
	South East	78	11.5 %	88.5%	
	North West	95	23.2 %	76.8%	
	East Midlands	39	23.1 %	76.9%	
	South West	51	21.6 %	78.4%	
	Scotland	53	11.3 %	88.7%	
	East of England	39	17.9 %	82.1%	
	Yorkshire & the Humber	55	12.7 %	87.3%	
IMD Decile (England only)	1	26	19.2 %	80.8%	0.36 (Small / Medium), 0.908
	2	45	15.6 %	84.4%	
	3	49	18.4 %	81.6%	
	4	52	15.4 %	84.6%	
	5	64	20.3 %	79.7%	
	6	52	17.3 %	82.7%	
	7	58	15.5 %	84.5%	
	8	57	24.6 %	75.4%	
	9	46	23.9 %	76.1%	
	10	50	14.0 %	86.0%	

* P < 0.05

Table S7: Descriptive statistics (row percentages) for the question "Did they provide information about bereavement support services? – Yes (during a follow-up call)". *P*-values are from chi-squared analysis of a contingency table. Effect sizes are estimated from Cohen's *h,* where $h = 0.2$: small effect, $h = 0.5$: medium effect, $h \geq 0.8$: large effect.

		Simple Logistic Regression			Generalised Linear Model		
		OR	95% LCI	95% UCI	OR	95% LCI	95% UCI
Who was it that died?	Partners	Reference Class					
	Parents	1.571	1.076	2.294	1.310	0.787	2.180
	Grandparent	4.944	2.411	10.139	9.332	2.033	42.841
	Sibling	2.649	1.059	6.625	6.387	1.150	35.473
	Child	0.706	0.230	2.167	1.357	0.217	8.483
	Other family member	5.808	2.618	12.883	7.716	1.860	32.014
	Colleague or friend	2.260	0.963	5.307	—	—	—
Where did they die?	In hospital	Reference Class					
	In their home	0.242	0.163	0.359	0.190	0.087	0.417
	In a hospice	0.435	0.220	0.859	0.887	0.341	2.310
	In a care home	1.053	0.655	1.693	1.181	0.622	2.242
	Other / Don't Know	0.104	0.023	0.475	0.864	0.080	9.339
Cause of Death	COVID (Confirmed or Suspected)	Reference Class					
	Non-Covid	0.318	0.233	0.435	0.601	0.361	1.000
Highest qualification	None / GCSEs	Reference Class					
	A-level / apprenticeship / ONC	0.782	0.468	1.306	1.217	0.609	2.431
	HND / University Degree / Postgraduate (etc)	0.833	0.546	1.273	1.111	0.610	2.022
Gender	Male	Reference Class					
	Female	1.188	0.734	1.924	0.640	0.311	1.319
	Other	6.000	0.688	52.313	0.000	0.000	—
Did you expect your loved one to die around this time?	Yes	Reference Class					
	No	3.288	2.131	5.072	1.404	0.661	2.982
	Don't Know	2.127	1.037	4.365	0.880	0.292	2.652

Note: The symbol "—" is given when results of simple linear regression or of the GLM became unreliable due to small sample sizes.

Table S8: Results for the Odds Ratio (OR) and associated 95% confidence intervals from simple logistic regression and a mixed-effects generalised linear model for the item "Unable to visit them prior to their death."

		Simple Logistic Regression			Generalised Linear Model		
		OR	95% LCI	95% UCI	OR	95% LCI	95% UCI
Who was it that died?	Partners	Reference Class					
	Parents	2.020	1.383	2.950	1.587	0.958	2.627
	Grandparent	1.991	1.052	3.768	0.960	0.313	2.947
	Sibling	1.523	0.629	3.686	1.240	0.277	5.545
	Child	0.426	0.130	1.397	0.094	0.009	0.982
	Other family member	1.998	1.014	3.938	1.335	0.429	4.157
	Colleague or friend	0.859	0.371	1.992	—	—	—
Where did they die?	In hospital	Reference Class					
	In their home	0.244	0.165	0.362	0.110	0.050	0.240
	In a hospice	0.391	0.198	0.773	0.557	0.226	1.371
	In a care home	1.710	1.008	2.901	1.390	0.700	2.757
	Other / Don’t Know	0.093	0.020	0.427	0.235	0.021	2.644
Cause of Death	COVID (Confirmed or Suspected)	Reference Class					
	Non-Covid	0.380	0.278	0.520	0.742	0.446	1.236
Highest qualification	None / GCSEs	Reference Class					
	A-level / apprenticeship / ONC	1.802	1.071	3.031	2.431	1.198	4.935
	HND / University Degree / Postgraduate (etc)	1.291	0.849	1.964	1.950	1.081	3.515
Gender	Male	Reference Class					
	Female	1.588	0.979	2.574	1.425	0.723	2.810
	Other	2.786	0.508	15.281	—	—	—
Did you expect your loved one to die around this time?	Yes	Reference Class					
	No	2.995	1.964	4.568	1.434	0.699	2.941
	Don’t Know	2.791	1.355	5.750	1.678	0.579	4.860

Note: The symbol “—” is given when results of simple linear regression or of the GLM became unreliable due to small sample sizes

Table S9: Results for the Odds Ratio (OR) and associated 95% confidence intervals from simple logistic regression and a mixed-effects generalised linear model for the item “Limited contact with them in last days of their life.”

		Simple Logistic Regression			Generalised Linear Model		
		OR	95% LCI	95% UCI	OR	95% LCI	95% UCI
Who was it that died?	Partners	Reference Class					
	Parents	1.933	1.323	2.826	1.561	0.897	2.714
	Grandparent	3.593	1.756	7.353	2.142	0.577	7.948
	Sibling	2.909	1.088	7.778	1.843	0.397	8.561
	Child	1.173	0.405	3.397	2.267	0.351	14.624
	Other family member	4.221	1.906	9.345	4.118	1.062	15.974
	Colleague or friend	1.939	0.814	4.621	—	—	—
Where did they die?	In hospital	Reference Class					
	In their home	0.231	0.157	0.340	0.212	0.099	0.454
	In a hospice	0.172	0.083	0.354	0.618	0.23	1.662
	In a care home	1.450	0.828	2.541	2.089	0.951	4.588
	Other / Don't Know	0.159	0.048	0.527	0.054	0	16.76
Cause of Death	COVID (Confirmed or Suspected)	Reference Class					
	Non-Covid	0.186	0.130	0.266	0.348	0.2	0.605
Highest qualification	None / GCSEs	Reference Class					
	A-level / apprenticeship / ONC	0.787	0.456	1.360	1.181	0.526	2.654
	HND / University Degree / Postgraduate (etc)	0.686	0.436	1.079	1.034	0.515	2.075
Gender	Male	Reference Class					
	Female	1.769	1.090	2.872	1.566	0.747	3.281
	Other	2.368	0.432	12.991	—	—	—
Did you expect your loved one to die around this time?	Yes	Reference Class					
	No	3.818	2.506	4.568	1.101	0.523	2.316
	Don't Know	1.705	0.840	5.750	0.525	0.168	1.643

Note: The symbol "—" is given when results of simple linear regression or of the GLM became unreliable due to small sample sizes.

Table S10: Results for the Odds Ratio (OR) and associated 95% confidence intervals from simple logistic regression and a mixed-effects generalised linear model for the item "Unable to say goodbye as I would have liked."

		Simple Logistic Regression			Generalised Linear Model		
		OR	95% LCI	95% UCI	OR	95% LCI	95% UCI
Who was it that died?	Partners	Reference Class					
	Parents	0.942	0.410	2.164	1.091	0.457	2.603
	Grandparents	0.544	0.170	1.743	0.545	0.104	2.853
	Sibling	0.370	0.091	1.512	0.596	0.065	5.484
	Child	0.778	0.091	6.677	0.356	0.034	3.776
	Other family member	0.796	0.202	3.133	0.993	0.128	7.72
	Colleague or friend	0.233	0.070	0.781	—	—	—
Where did they die?	In hospital	Reference Class					
	In their home	0.790	0.387	1.614	0.561	0.189	1.665
	In a hospice	—	—	—	2.642	0.289	24.113
	In a care home	0.592	0.266	1.314	0.315	0.118	0.838
	Other / Don't Know	0.779	0.097	6.235	1.771	0.005	649.828
Cause of Death	COVID (Confirmed or Suspected)	Reference Class					
	Non-Covid	0.523	0.275	0.995	0.66	0.273	1.592
Highest qualification	None / GCSEs	Reference Class					
	A-level / apprenticeship / ONC	2.444	0.939	6.362	1.631	0.564	4.721
	HND / University Degree / Postgraduate (etc)	2.326	1.153	4.693	1.918	0.794	4.635
Gender	Male	Reference Class					
	Female	2.496	1.185	5.255	2.292	0.906	5.799
	Other	—	—	—	—	—	—
Did you expect your loved one to die around this time?	Yes	Reference Class					
	No	2.112	1.071	4.167	1.179	0.397	3.502
	Don't Know	2.665	0.576	12.337	1.812	0.287	11.428

Note: The symbol "—" is given when results of simple linear regression or of the GLM became unreliable due to small sample sizes

Table S11: Results for the Odds Ratio (OR) and associated 95% confidence intervals (i.e., 95% LCI and 95% UCI) from simple logistic regression and a mixed-effects generalised linear model for the item "Restricted funeral arrangements."

		Simple Logistic Regression			Generalised Linear Model		
		OR	95% LCI	95% UCI	OR	95% LCI	95% UCI
Who was it that died?	Partners	Reference Class					
	Parents	0.421	0.266	0.666	0.292	0.158	0.539
	Grandparents	0.304	0.155	0.599	0.159	0.051	0.493
	Sibling	0.294	0.117	0.737	0.114	0.025	0.514
	Child	0.452	0.143	1.425	0.718	0.103	5.021
	Other family member	0.246	0.121	0.501	0.092	0.028	0.297
	Colleague or friend	0.308	0.128	0.742	—	—	—
Where did they die?	In hospital	Reference Class					
	In their home	0.804	0.547	1.183	1.371	0.664	2.831
	In a hospice	1.412	0.648	3.079	2.734	0.952	7.852
	In a care home	0.798	0.496	1.284	0.81	0.426	1.541
	Other / Don’t Know	0.284	0.091	0.884	—	—	—
Cause of Death	COVID (Confirmed or Suspected)	Reference Class					
	Non-Covid	0.483	0.348	0.670	0.439	0.261	0.739
Highest qualification	None / GCSEs	Reference Class					
	A-level / apprenticeship / ONC	0.959	0.558	1.648	1.052	0.523	2.115
	HND / University Degree / Postgraduate (etc)	0.950	0.608	1.484	1.597	0.867	2.94
Gender	Male	Reference Class					
	Female	1.238	0.752	2.038	1.439	0.729	2.841
	Other	1.522	0.276	8.378	—	—	—
Did you expect your loved one to die around this time?	Yes	Reference Class					
	No	1.543	1.017	2.340	1.211	0.598	2.454
	Don’t Know	0.867	0.427	1.763	0.645	0.241	1.725

Note: The symbol “—” is given when results of simple linear regression or of the GLM became unreliable due to small sample sizes.

Table S12: Results for the Odds Ratio (OR) and associated 95% confidence intervals (i.e., 95% LCI and 95% UCI) from simple logistic regression and a mixed-effects generalised linear model for the item “Social isolation and loneliness.”

		Simple Logistic Regression			Generalised Linear Model		
		OR	95% LCI	95% UCI	OR	95% LCI	95% UCI
Who was it that died?	Partners	Reference Class					
	Parents	1.134	0.700	1.835	0.91	0.504	1.643
	Grandparents	0.744	0.354	1.564	0.329	0.104	1.04
	Sibling	0.668	0.242	1.843	0.265	0.055	1.289
	Child	0.648	0.193	2.183	2.068	0.207	20.613
	Other family member	1.120	0.472	2.655	0.948	0.234	3.842
	Colleague or friend	0.445	0.180	1.099	—	—	—
Where did they die?	In hospital	Reference Class					
	In their home	0.749	0.476	1.178	1.111	0.51	2.422
	In a hospice	1.757	0.604	5.115	2.409	0.701	8.278
	In a care home	0.756	0.433	1.321	0.968	0.45	2.084
	Other / Don't Know	0.341	0.108	1.072	0.206	0.025	1.671
Cause of Death	COVID (Confirmed or Suspected)	Reference Class					
	Non-Covid	0.479	0.321	0.716	0.465	0.254	0.852
Highest qualification	None / GCSEs	Reference Class					
	A-level / apprenticeship / ONC	1.646	0.898	3.017	1.73	0.836	3.581
	HND / University Degree / Postgraduate (etc)	1.811	1.116	2.937	2.833	1.504	5.337
Gender	Male	Reference Class					
	Female	2.101	1.232	3.580	2.116	1.059	4.227
	Other	0.601	0.124	2.907	—	—	—
Did you expect your loved one to die around this time?	Yes	Reference Class					
	No	1.334	0.819	2.175	1.29	0.598	2.785
	Don't Know	1.129	0.480	2.656	1.092	0.351	3.396

Note: The symbol "—" is given when results of simple linear regression or of the GLM became unreliable due to small sample sizes

Table S13: Results for the Odds Ratio (OR) and associated 95% confidence intervals (i.e., 95% LCI and 95% UCI) from simple logistic regression and a mixed-effects generalised linear model for the item "Limited contact with other close relatives or friends."
